# Forest soil biotic communities show few responses to wood ash applications at multiple sites across Canada

**DOI:** 10.1038/s41598-022-07670-x

**Published:** 2022-03-09

**Authors:** Emily Smenderovac, Caroline Emilson, Teresita Porter, Dave Morris, Paul Hazlett, Amanda Diochon, Nathan Basiliko, Nicolas Bélanger, John Markham, P. Michael Rutherford, Ken van Rees, Trevor Jones, Lisa Venier

**Affiliations:** 1 Great Lakes Forestry Centre, Sault Ste. Marie, Natural Resources Canada P6A 2E5 Canada; 2 Centre for Northern Forest Ecosystem Research, Ontario Ministry of Northern Development, Mines, Natural Resources and Forestry, Thunder Bay, P7E 2V6 Canada; 3 Lakehead University, Thunder Bay, P7B 5E1 Canada; 4 Laurentian University, Sudbury, P3E 2C6 Canada; 5 Université TELUQ, Québec City, G1K 9H6 Canada; 6 University of Manitoba, Winnipeg, R3T 2N2 Canada; 7 University of Northern British Columbia, Prince George, V2N 4Z9 Canada; 8 University of Saskatchewan, Saskatoon, S7N 5B5 Canada

**Keywords:** Biodiversity, Community ecology, Forestry, Microbial ecology, Molecular ecology, Boreal ecology, Community ecology, Forestry, Microbial ecology, Molecular ecology

## Abstract

There is interest in utilizing wood ash as an amendment in forestry operations as a mechanism to return nutrients to soils that are removed during harvesting, with the added benefit of diverting this bioenergy waste material from landfill sites. Existing studies have not arrived at a consensus on what the effects of wood ash amendments are on soil biota. We collected forest soil samples from studies in managed forests across Canada that were amended with wood ash to evaluate the effects on arthropod, bacterial and fungal communities using metabarcoding of F230, 16S, 18S and ITS2 sequences as well as enzyme analyses to assess its effects on soil biotic function. Ash amendment did not result in consistent effects across sites, and those effects that were detected were small. Overall, this study suggests that ash amendment applied to managed forest systems in amounts (up to 20 Mg ha^−1^) applied across the 8 study sties had little to no detectable effects on soil biotic community structure or function. When effects were detected, they were small, and site-specific. These non-results support the application of wood ash to harvested forest sites to replace macronutrients (e.g., calcium) removed by logging operations, thereby diverting it from landfill sites, and potentially increasing stand productivity.

## Introduction

Canada is increasingly incorporating bioenergy into provincial energy strategies. Historically, manufacturing by-products have been a common source of energy for the timber and pulp and paper industries and, more recently, wood/biomass thermal plants are being established explicitly for electricity and heat production. A by-product of burning these waste materials for heat and power is wood ash, which is typically disposed of in landfills^1^. Application of wood ash in forests could result in some economic benefit, by reducing or eliminating the need to landfill ash. Wood ash has the potential to maintain or enhance site productivity, by possibly supplementing or reducing existing commercial fertilizer and lime use^2^. Silvicultural harvesting practices can cause increased acidity and calcium limitation in soils through removal of tree biomass. In some locations, these effects are mitigated through addition of calcium based supplements such as lime^3^.

Wood ash has many properties that could increase soil quality. Wood ash is typically alkaline, and so it can increase the pH of soils which increases microbial activity and nutrient availability^4,5^. It is also high in calcium and magnesium, elements crucial for plant growth and health^6^. In some cases, wood ash amendment can increase soil carbon pools as well as increase tree growth^7^. Emilson et al.^8^ observed increased growth of jack pine trees with ash addition. There are additional possible downstream benefits that should also be considered, such as the alleviation of calcium limitation in watersheds that have been affected by acid rain^9^ and, some studies have recorded increases to amphibian and earthworm assemblages^10,11^. These alterations have the potential to influence other soil taxa. Ash can contain toxic metals such as cadmium or manganese, and the application process can create physical disruption to soil surfaces and vegetation that may negatively affect soil communities and processes^12,13^. On the other hand, ash has been demonstrated to mitigate effects of pollutants, such as Cadmium and antibiotics, on fungal and bacterial community structure and growth^14,15^.

Soil arthropod, bacterial and fungal communities are known to be responsive to differences in moisture, pH and calcium levels^16^. While harvest disturbance alters moisture, pH and calcium which results in stark changes to microbial community composition from uncut forest systems, increases to harvesting intensity can be small and undetectable^17^. The effects of wood ash amendment are likely also small; a recent study of sites across Canada showed inconsistent responses in soil quality metrics due to wood ash amendment^18^. In general, reported microbial responses to ash application are inconsistent, and changes in mineral layers take longer than those in organic layers^19–22^. Though the potential benefits of ash use are great, a barrier to ash use is that it is generally still classified as an industrial waste product, and attaining regulatory approval remains a confusing and difficult process^1,23^.

The current body of literature typically consists of case studies that evaluate and identify soil biota responses and changes due to wood ash amendment in specific stands and silvicultural systems^5,19^. Comparison of these studies can be challenging due to methodological differences between studies (i.e., metabarcoding has not been widely applied, and so use of various methods to assess community composition have been employed such as PLFA, microbial respiration, etc.). These site-specific results make it difficult to extrapolate the effects of ash amendment to systems at large, and has impeded the classification of ash as a safe and viable soil amendment. We used a collaborative network (AshNet; a cross-Canada network assessing the effects of wood ash amendment in harvested forests), with standardized sample collections, data sharing and archiving, that allows for the ability to study several sites along a gradient of ash amendment treatments and ecosystems across Canada^24^.

Along with site specific studies, literature often focuses on one or two components of a biological community (e.g., 16S targeted sequencing for bacterial communities, ectomycorrhizal communities or arthropod communities)^19,25–27^. As sequencing technology continues to become more cost-effective, multi-taxa studies can be used to provide data for more comprehensive evaluation of the soil biotic community. By collectively examining multiple groups of soil taxa, along with a functional assessment associated with community change, a more holistic analysis of treatment effects within soils can be achieved. Examining enzyme activities is a well-established methodology for studying a “snapshot” of the in-situ metabolic activity of soil biotic communities. N-acetylglucosaminidase and phosphatase activity are two such enzymes. N-acetylglucosaminidase serves as a measure of nitrogen scavenging from bacterial and fungal cells, as well as insect exoskeletal material, whereas phosphatase serves as a measure of phosphate scavenging from organic molecules^28,29^. Included along with soil biotic information, these measures can provide evidence of nitrogen or phosphorus excess or limitation and the efficiency of nutrient cycling processes within the system. Targeted metabarcoding provides a robust measure of the whole community assemblage, including the organisms past and present^30^.

This study utilizes a multi-metric analysis of soil community structure and function within eight AshNet sites to determine if there are any measurable impacts on the ecology of forest soil systems. This study assesses what effects wood ash amendment has on soil biota composition and function; and, explores whether those changes are affected by site characteristics, ash characteristics or, the amount of ash added. We anticipate there will be minimal or inconsistent effects of wood ash amendment on the soil biotic community composition or functional enzyme responses when applied to managed forested sites. We expect that any detectable differences (i.e., community shifts or functional changes) will be infrequent, site-specific, small, confined to organic soil horizons^21^, and, correspond to higher ash application rates (i.e., supporting that wood ash amendment does not create large changes to soil community structures).

## Methods

### Experimental conditions

**Figure 1:**
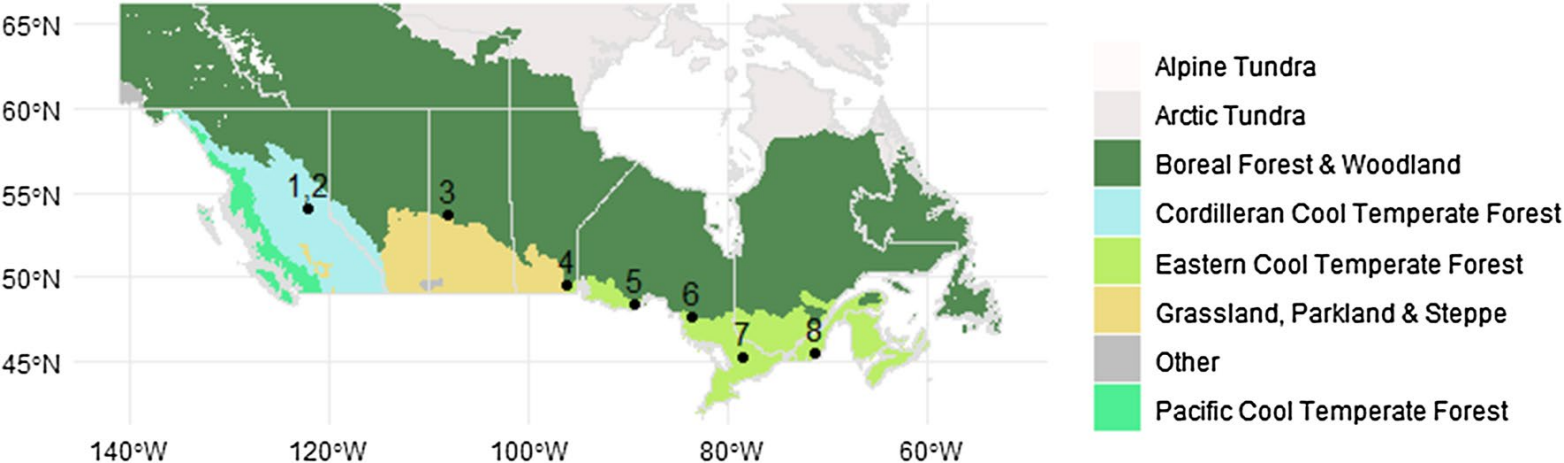
Location of the wood ash study sites across Canada. Site names from left to right include (1) Aleza Lake N, (2) Aleza Lake S, (3) Mistik, (4) Pineland, (5) 25th Sideroad, (6) Island Lake, (7) Haliburton, (8) Eastern Townships Maple. The map is shaded by major forested Vegetation Zones of Canada (https://open.canada.ca/data/en/dataset/22b0166b-9db3-46b7-9baf-6584a3acc7b1/resource/58fdcc95-5879-4271-92aa-87ca28affa7d) and was constructed in R using ggplot2 with downloaded shapefiles^43,44,46^. Sites used in this study are located on Cordilleran Cool Temperate forest , Boreal Forest , and, Eastern Cool Temperate Forest .

In order to assess the potential biodiversity effects of wood ash amendment in forestry operations, soil samples from eight sites were assessed from the AshNet Network^31^ (Table 1, Fig. 1). Additional details about these sites are described in Emilson et al.^31^ To ensure that a range of conditions were assessed, sites from different provinces were selected for barcoding and functional enzyme analyses: Aleza Lake (N & S) in British Columbia; Mistik (Burness) in Saskatchewan; Pineland in Manitoba; 25th Sideroad, Haliburton, and, Island Lake in Ontario; Eastern Townships-Sugar Maple in Quebec. These sites represent multiple ecozones (i.e., Montane Cordillera, Boreal Plains, Boreal Shield, and Mixedwood Plains)^32^. Dominant tree species and soil characteristics vary accordingly due to ecozone and location. Additionally, different harvesting systems were used, ranging from single tree selection to more intensive practices of clear cutting and disc-trenching. See Table 1 for a summary of site characteristics. At each site, a single application of wood ash was applied. There was a range of stand ages at the time of application: directly after harvest, to as long as 24 years after harvest. Each site contained either paired control and treatment plots, or block designs with treatments and controls included in each block. Sites also had a variety of wood ash types and application amounts, including fly and bottom ash from different sources and ranging from 5 to 20 dry tonne equivalent per hectare.

**Table 1:** Site and climate characteristics associated with the eight AshNet sites used in this study.

Site code	Site name	Ash types	Ash application (Mg/ha)	Ash Calcium additions (kg/ha)	Years between Ash application and sampling	Harvesting	Province	Soil type	Stand type	May mean monthly maximum temperature (C)	Precipitation of wettest quarter (mm)
ALN	Aleza Lake N	Bottom ashes from gasifier and single boiler	0, 5	0, 335, 970	2	Clearcut	British Columbia	Gray Luvisolic with Luvic Gleysolic	18 year old hybrid spruce plantation	17.18	206
ALS	Aleza Lake S	Bottom ashes from gasifier and single boiler	0, 5	0, 335, 970	2	Clearcut; Broadcast burn	British Columbia	Gray Luvisolic with Luvic Gleysolic	24 year old hybrid spruce plantation	17.02	208
MSK	Mistik (Burness)	Bottom ash from a single boiler	0, 1, 5	0, 16.84, 84.2	22	Clearcut full tree + distrenched	Saskatchewan	Orthic Gray Luvisol	White Spruce planted	16.00	204
PLD	Pineland	Mixed ash from a single boiler	0, 1.5	0, 273.495	2	Clearcut whole tree	Manitoba	Brunisols	Jack Pine	18.02	269
SRD	25th sideroad (Lakehead)	Fly ash from a single boiler	0, 1, 10	0, 170.28, 1702.8	5	Former Nursery, tilled	Ontario	Orthic Eutric Brunisols	black and white spruce	15.97	243
ILK	Island Lake	Bottom ash from a single boiler	0, 0.7, 1.4, 2.8, 5.6	0, 50, 100, 200, 400	6	Clearcut full tree	Ontario	Eluviated Dystric Brunisols	Jack Pine	16.09	292
HLB	Haliburton	Bottom, fly ash from a single boiler	0, 1, 4, 8	0, 43.6, 174.4, 348.8, 101.1, 404.4, 808.8	4	Single Tree Selection	Ontario	Orthic or Eluviated Dystric Brunisols	Mixed deciduous	17.80	317
ETM	Eastern Township Sugar Maple	Bottom ash from a single boiler	0, 20	0, 3516	2	Selection cut	Quebec	Orthic Humo-Ferric / Ferro-Humic Podzols	Mixed deciduous	16.80	382

### Soil sampling

All soil samples were collected in June or July 2017 or 2018. Due to logistical constraints, aside from ETM, HLB and ILK where two replicates were sampled from each block, one sample was collected from each block within each site that was sampled. The fresh litter-moss horizon (L), the fermentation-humus (FH horizons) and upper mineral soil (MIN, 0–10 cm) were sampled separately. When L and FH horizons could not be effectively separated during sampling, a pooled sample was taken (LFH). L and FH horizons were sampled from a 15 × 15 cm frame, after which mineral soil was collected in a 5 cm diameter PVC pipe to a depth of 20 cm. PVC pipes were sanitized with bleach or concentrated alcohol before sampling was performed. All sampling was completed with gloves to reduce cross-contamination. Samples were immediately frozen at – 20 °C after sampling and held at that temperature until analysis. All samples were shipped to the Great Lakes Forestry Centre (Sault Ste. Marie) where soils were homogenized, and subsamples were aseptically taken for enzyme and metabarcoding analyses. One homogenized sample was taken for each treatment at each block of each site.

### Enzyme analyses

Enzyme analyses were performed for the ecologically important enzymes N-acetylglucosaminidase (NAG) and phosphatase (PHOS). All enzyme activities were performed in 96-well plates under controlled conditions using 4-methylumbelliferone-fluorescence tagged substrates (pH 5 and room temperature) and measured with a BioTek Synergy H1 Hybrid spectrophotometer/fluorometer. Incubation times were based on time-trials conducted on a subset of samples. Samples were stored at – 20 °C prior to analysis via existing protocols^33,34^.

### Metabarcoding

DNA extractions were performed on homogenized bulk soil samples using Qiagen DNEasy Powersoil extraction kits. Extracted DNA was then amplified using primers targeted for specific groups of organisms (Supplemental Materials, Tables S1, S2). Paired-end sequencing for Arthropods (F230, 18S), Fungi (18S and ITS) and Bacteria (16S) were performed on the Illumina MiSeq platform in the Hajibabaei lab at the Centre for Biodiversity Genomics, University of Guelph. A total of ~ 265 million paired-end reads were generated from 4 amplicons (Supplemental Materials Table S3). Data was processed into ASVs (Amplicon Sequence Variants) using the MetaWorks pipeline v1.4.0^35^. Sequence retention details are provided Tables S3 and S4 of the Supplemental Materials. Samples with less than 1000 reads were removed. Taxonomic assignments with at least 80% accuracy were retained using the bootstrap cutoff values for the ITS, COI and 18S classifiers genus level for 300 bp reads. We used a cut off value of 80% for the 16S region as recommended by the RDP classifier documentation. Taxonomy of 16S data was assigned using the RDP 2.1.3 database included with MetaWorksv1.4.0. Taxonomic assignment of 18S sequences was completed with SILVA 138 SSURef Nr99 trained to work with the RDP 2.13 classifier^36^. F230 sequences were classified using the CO1 Classifier v4^37^. ITS sequences were classified with the UNITE v8.2 ITS reference set database^38,39^. Functional guilds were assigned through FAPROTAX (16S), FUNGuild (ITS) or using the BETSI database (F230)^40–42^. A summary of the percent of ASVs identified to Genus using the appropriate cut-off values, and the subsequent successful assignment of functional guild with those taxonomy are provided in Table S5 of the Supplemental Materials.

### Site and ash characteristics

Several variables were included in the dataset to represent site and ash characteristics. These data were acquired through collaborators or performed at Great Lakes Forestry Centre analytical facilities. Stand age (age of stand at ash application), dominant tree species (most common tree species type), precipitation seasonality (a coefficient that represents how much variation in precipitation there is throughout the year), precipitation of the wettest quarter (how much rain occurred in the wettest quarter of the sampling year), and May mean monthly maximum temperature (maximum may temperature during the year of sampling) represented site differences in models. Stand age and dominant tree species were selected as variables because there were common characteristics across sites, where other variables (e.g. soil class), corresponded exactly with site differences. May monthly temperature and precipitation of the wettest quarter (summer, in these sites) were selected as they represented differences in temperature and precipitation across the sites in a uniform period before the sampling time (June-July). Precipitation seasonality was included because it gave an indication of whether there was variation in precipitation across the year. Climate variables were produced by the climate models of McKenney et al.^43^ Total soil carbon and total soil nitrogen data were calculated via TOC/TON analysis as described in Joseph et al.^18^ Differences between control and treatment soil samples were used as explanatory parameters within models.

Ash characteristics utilized in the analysis included the calcium, phosphorus and sodium quantities added to the sites via ash addition. These variables were selected from a larger set of ash chemical parameters to serve as representatives in the analysis due to completeness, high correlation with other parameters in the dataset and their ecological relevance to nutrients and metal toxicity, importance as a limiting nutrient and toxicity respectively.

### Statistical analyses

ANOVA mixed effects models were performed in R to assess whether ash amendment or amount of amendment affected enzyme activity with the different soil horizons and the sites as random effects. Additional fixed effects ANOVA were performed on the same variables with interaction terms, leading to the decision to investigate site-level responses with pairwise comparisons^44^.Differences in enzyme response between treatments and controls were not normally distributed among blocks and were assessed by Wilcoxon-tests for each parameter and significance was discussed in the context of a Bonferonni corrected $\alpha$ of 0.05. Wilcoxon tests were performed on the difference in enzyme response within blocks to remove the influence of within-site spatial variation. Difference in enzyme activities were also subjected to multiple regression. Models were determined using forward selection with site characteristics (i.e., stand age, dominant tree species, age of stand at ash treatment, precipitation seasonality, precipitation of the wettest quarter, May mean monthly maximum temperature, and, differences between control and treatment soil for total soil carbon and soil total nitrogen) and, components supplied by ash (i.e., total applied calcium, sodium, phosphorus) as input. Organic (ORG—L, FH and LFH) and mineral (MIN 0–10 cm) soil horizons were assessed in separate models.

Metabarcoding data were analysed in R using data transformation functions found in the tidyverse package, ALDEx2 for compositional analyses and vegan for diversity analyses using a more standard approach^44–47^. For standard diversity analyses, bacterial (16S) communities were assessed as relative abundance using Bray–Curtis distance, and all other metabarcoding communities were assessed as presence/absence matrixes using Jaccard distances. Alpha diversity metrics (Shannon, inverse-Simpsons and richness) for each metabarcode were subjected to mixed effects ANOVA with interactions,including whether ash was applied and, ash addition amount with site and soil horizon as random effects to test the effect of ash amendment after accounting for site and horizon differences. In order to test whether there were effects that were masked by variation between blocks, we performed Wilcoxon tests of the difference in diversity metrics between controls and treatments within each block, which were evaluated at a Bonferroni corrected alpha. Compositional analyses were performed on data transformed using centered log ratio (clr) and consisted of PCA and RDA with ash parameters (i.e. total applied calcium, phosphorus, and, sodium) as constraining variables. ALDEx2 was used to assess the compositional change of individual taxa. ALDEX glms were performed for each targeted amplicon (i.e., 16S, 18S, ITS, F230) with site characteristics (i.e., Stand age, dominant tree species, precipitation seasonality, precipitation of the wettest quarter, and May mean monthly maximum temperature, soil total carbon, soil total nitrogen) and, components supplied by ash (i.e., total applied calcium, sodium, phosphorus) as input, mineral soil horizons and organic soil horizons were assessed in separate models. ALDEX pair-wise analyses were performed on each targeted amplicon, for each treatment–control pairing with separate models for organic and mineral soil horizons to identify any ASV, functional group or genus with statistically significant change in composition.

A general summary of all models run for the analysis can be found in the Supplemental Material (Table S6).

## Results

### Enzyme analyses

**Figure 2:**
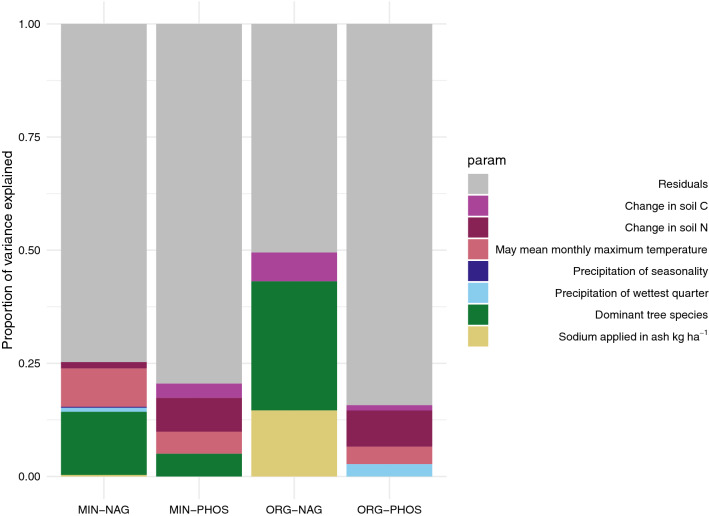
Variance of difference explained by linear modelling parameters for models of the difference in enzyme activities (NAG = N-acetylglucosamine, PHOS = phosphatase) between ash amended soils and controls for mineral (MIN = 0–10 cm of mineral soil) and organic (ORG = L, FH and LFH) soil horizons. Parameters in models are displayed in different colours: Residuals , Change in Soil Carbon , Change in Soil Nitrogen , May mean monthly maximum temperature , Precipitation of Seasonality , Precipitation of wettest quarter , Dominant tree species , Sodium applied in ash kg ha^−1^ .

Mixed effects ANOVA results showed that ash addition did not significantly explain any difference in either NAG or PHOS activity after accounting for site and soil horizon (*p* < 0.05) (Supplemental Materials, Tables S7-S10). When linear modelling of the difference in activity between treatments and controls was performed, all fitted models were significant (*p* < 0.05), with adjusted R^2^ values ranging from 0.05 to 0.48 explaining variance between 0.08 and 0.5. The linear model which explained a high amount of variance (0.5) was the model for NAG activity in organic horizons, for which most of the variance was explained by stand composition. There was no significant (*p* < 0.05) direct influence of ash application on NAG or PHOS activity in mineral soils. More of the variance in enzyme activity differences across sites in organic horizons to ash addition was explained by the dominant tree species in the stand, climate related variables (precipitation, temperature), and carbon differences in the soils, than because of ash sodium additions (Fig. 2). While precipitation seasonality and may mean temperature were also incorporated into models for enzyme response in organic horizons, parameters related to soil characteristics (increased N or C as compared to controls) or calcium and sodium application rates were found to be significantly influencing changes in NAG and PHOS activity.

**Figure 3:**
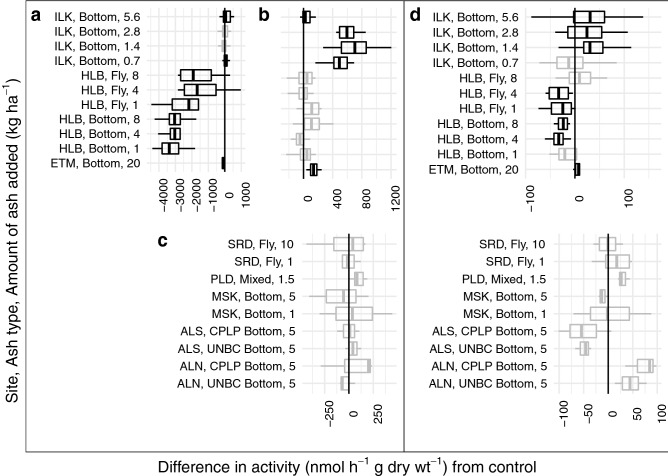
Differences in *N*-acetyl-glucosamine activity between treatments and controls.Results significantly different than zero as determined by Wilcoxon-test are shown with  (Bonferonni corrected *p* < 0.05) or  (*p* < 0.05), results that were not significant are shown with . Panels show different soil horizons a = L, b = FH, c = LFH, d = 0–10 cm of mineral soil.

**Figure 4:**
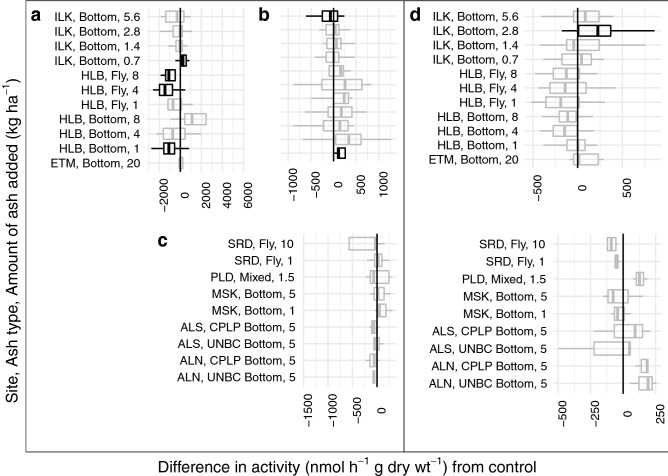
Differences in phosphatase activity between treatments and controls. Results significantly different than zero as determined by Wilcoxon-test are shown  (*p* < 0.05), results that were not significant are shown with . Panels show different soil horizons a = L, b = FH, c = LFH, d = 0–10 cm of mineral soil. No responses were significant at a Bonferroni corrected $\upalpha$.

Statistically significant responses of enzyme activity to ash amendment, as assessed with Wilcoxon tests against a Bonferonni corrected alpha, were only found in the ILK site. NAG activities were significantly increased for three ash application rates in the Island Lake FH horizon (Fig. 3). Significant effects at an uncorrected $\alpha$ of 0.05 were found in ETM, HLB, and ILK sites. Island Lake had one amendment rate that resulted in significantly increased MIN horizon PHOS activity (Fig. 4). There was decreased PHOS activity for different levels of ash amendment in L and MIN horizons at HLB sites and one amendment rate in the MIN horizon at the ILK site, and increases in PHOS activity for different amendment rates in the L and MIN horizons at the ILK sites, and in the FH horizon of the ETM site (Fig. 4). NAG appeared to have non-significant trends of increased activity in mineral soils of ALN, ILK and PLD sites, but decreased activity in ALS, all HLB amendment rates and MSK (Fig. 3). There were non-significant trends of decreased NAG activity in the L horizon of the HLB site, while ILK showed non-significant trends of increased NAG activity in MIN and FH horizons. Across sites, there was no consistent relationship between the activity changes of different soil horizons. The direction of response for both NAG and PHOS activity were inconsistent across sites and amendment rates.

### Biodiversity analyses

Mixed effects ANOVA results showed that ash addition did not affect diversity for any metabarcode group after accounting for site and soil horizon (*p* < 0.05). While there were some significant changes in alpha diversity metrics pair-wise Wilcoxon tests (at a Bonferroni-corrected $\alpha$ of 0.05), no site exhibited changes in alpha diversity across all assessed groups, for all metrics across all levels of grouping assessed (ASV, functional, genus). Most of the significant differences were at ASV or genus level and significant results were infrequent and inconsistent in direction (Supplemental Materials Tables S11–S12, Fig. S1, Fig S2).

**Figure 5:**
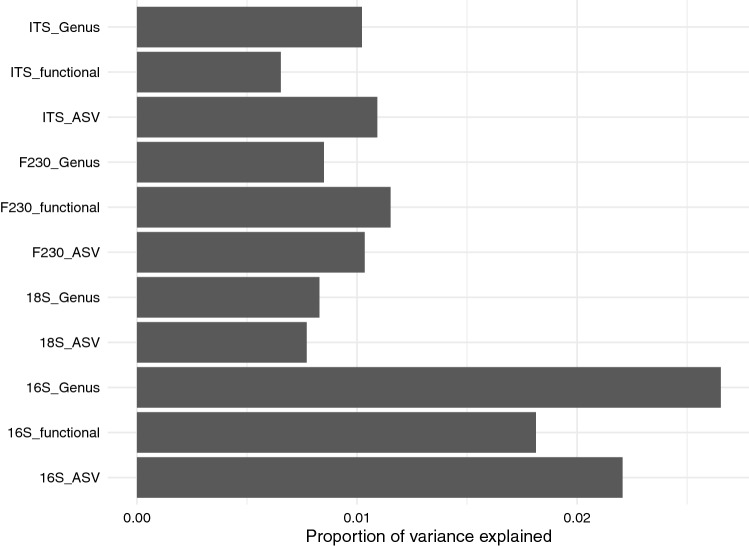
Proportion of variance explained by RDA axes constrained by the ash amendment parameters of applied calcium kg ha^−1^, applied phosphorus kg ha^−1^, and, applied sodium kg ha^−1^, after controlling for site and soil horizon. Model names are described as the targeted gene (16S, 18S, ITS, or, F230), summarization level (ASV, genus, or functional groupings).

PCA of clr transformed values suggested that the variance in the community compositions are not responding to strong, simple gradients (i.e., small proportions of variance were explained across many principal components). The 16S dataset, had 23% of the compositional variance explained in the first two principal components, and none of the eukaryotic groups (18S, ITS, F230) had more than 20% of the compositional variance explained in the first two principal components (Supplementary Fig. S3). When the influence of site and soil type were accounted for, RDA constrained with ash-related parameters (applied ash calcium, sodium and phosphorus) explained less than 3% of compositional variance (Fig. 5). Subsequent visual investigation of these RDAs showed that these relationships were not consistent across sites (Supplemental Materials Figs. S4–S7). This supported the use of pairwise analyses to investigate the impact of ash amendment by direct comparison of treatments and controls.

**Figure 6:**
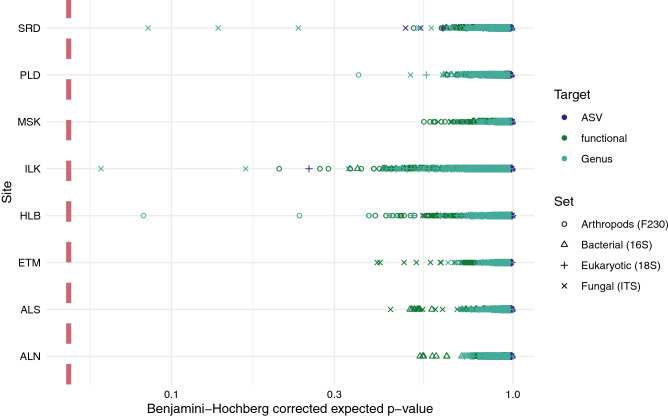
Benjamini–Hochberg corrected significance of pairwise ALDex2 from each control-treatment pairing for each site and metabarcoding target in the study at three levels of organization. Significance (*p* < 0.05) is shown on the graph as a light coloured dashed line. Colour and shape are used to differentiate the targeted sequence (Arthropods (F230) , Bacterial (16S) , Eukaryotic (18S) , Fungal (ITS) ) and grouping level (functional, Genus  and ASV). used for each test.

ALDEx2 glm models found one ericoid mycorrhizal fungus, *Distigmoptera* (a genus of herbivore beetles), and four other arthropod ASVs to be significantly associated with ash related parameters after Benjamini–Hochberg corrections in ORG horizons and one *Archaeorhyzomyces* (root endophyte) in the MIN horizon, no other groups were significantly associated with ash related parameters (Supplemental Materials Fig. S8). Subsequently, ALDEx2 pairwise models did not reveal any statistically significant differences between control and treatment samples for any site after the Benjamini–Hochberg corrections (Fig. 6). GLMs of gain/loss comparisons between controls and treatments within blocks did find six groups that were significantly associated with a higher incidence of presence or absence of that group across ash treatments in ORG horizons (Supplemental Materials Fig. S9). These groups corresponded to mycorrhizae (*Rhizoschyphus*, Sebacinales), saprotrophs (*Toxicocladosporium*), ciliates (*Uroleptus*) or unidentified. Subsequent analysis suggested these relationships were site specific and inconsistent, with most sites having small increases or decreases in gain or loss of a particular group (e.g., Losses of *Uroleptus* at the SRD site and gains at ALN and ALS sites, with little change elsewhere) (Supplemental Materials Fig. S10). Taken together these results show that the organisms may have had significant changes in their compositional importance, but were generally present regardless of treatment.

## Discussion

There are reports of changes to soil functional and community composition due to the addition of ash throughout the literature. However these lack consistency in the amount of amendment required to elicit a response and the direction of response^19,22,48–50^. Comparing the results of these studies is further complicated by methodological differences between them. Though sites and treatments were varied, the standardized data collection and analysis provided by AshNet allowed for highly comparable results amongst sites. As far as we are aware, AshNet is the first study to analyze experiments at multiple sites across Canada in a multi-targeted metabarcoding analysis of the effect of ash amendment on forest soil biotic communities. The infrequent and inconsistent responses across the many parameters in this study showed that soil arthropod, bacterial and fungal community responses to one-time wood ash additions are likely to be site-specific and small in comparison to within-site variation.

The lack of difference in metabarcoding community structures resulting from wood ash addition demonstrate very little evidence of direct effects of wood ash amendment on overall community composition for arthropod, bacterial or fungal communities (Figs. 5, 6). There could be some biodiversity implications to this, as we saw that alpha diversity metrics had some site-specific differences as well for specific targeted groups. The lack of demonstrable community differences, as well as the lack of consistency within-sites and between sites for alpha diversity and compositional tests confirms that we cannot make generalized conclusions about the effects of ash amendment on community assemblages. This study is not unique in this finding, as other studies have found soil communities to be unresponsive to ash addition^51–56^. In other studies where community-level or functional responses were detected, they were limited to higher amendment rates (9 Mg ha^−1^ or higher) and upper organic layers of soil^22,50,57^. The magnitude of Bray–Curtis or Jaccard distance differences and low explanatory power of constrained RDA axes also supports that ash amendment did not result in particularly large shifts in community structure. The infrequent effects as seen in this study and others within the AshNet network suggest that negative effects on biological community structure and function are minimal^18,58,59^. Other studies imply that the risk of damaging soil biotic function associated with heavy metal increases are low, and likely to be mitigated by the high calcium and magnesium concentrations in ash^6,60^. High calcium and magnesium concentrations in ash have more potential to alleviate metal stresses as many of these ions are in the forms of oxides and hydroxides, which increase pH and lower metal availability^4^. Observed responses were also in line with the known soil chemical response to ash addition^18^. In general, chemical response to ash addition is variable, with reports of increased soil quality after ash addition^6,21,48^, some where minimal or no changes were detected or, the changes were limited to specific ash and amendment rates^6,59^. So it is not unexpected that we also saw variability among site functional responses, which are, in part, regulated by soil chemistry. Given that results do not indicate frequent and consistent effects of ash on soil biota, the benefits of ash can likely be explored without large impacts to soil biodiversity. Emilson et al.^8^ found models including ash addition amounts explained some variance (~ 18%) in short term tree growth response on AshNet sites, and much of the variance was attributed to species specific response differences. Additional growth benefits may be more apparent in longer-term time scales, or in species with faster growth, such as *Acer* species^6^. There may be other value-added benefits in response to ash addition, such as increased nutrient availability that were not assessed in their study. Hope et al.^2^ estimated that ash amendment only becomes preferable to landfilling when an additional $15 CAD/tonne in benefits can be achieved. The site and species dependence of tree growth responses mean that the efficacy of ash amendment needs to be assessed before it is applied on operational scales.

In general, enzyme activities and functional community analysis confirms that ash amendment did not have any consistent measurable effects on functional attributes in soil biotic communities. While there were some positive and negative trends, and some shifts in functional community attributes, these results were infrequent, inconsistent and site specific. While differences between controls and treatments were shown in multiple regression models, models of the difference in enzyme response supported that the site characteristics had a greater impact on whether ash amendment changed NAG or PHOS activity than the specific chemical additions of ash (Fig. 2). That the ash signal was not also observed in the mixed-model ANOVA tests or the fixed effects ANOVA with interactions, which did not account for block effects, showed that these responses in enzyme activity were small enough to be masked by in-site spatial variation or very site-dependent (Supplemental Materials Tables S7–S10). The observed differences in enzyme activity, as demonstrated via pairwise Wilcoxon tests, were not linked to functional activities present in the metabarcoding communities (e.g., ILK treatments had significant increases in N-acetylglucosamine activity in the L horizon, but no corresponding shift in functional community structure or alpha-diversity metrics) (Fig. 3, Supplemental Materials Figs. S4–S7). This indicates that these changes are likely temporary adaptations rather than persistent selective forces that would result in differences corresponding particular functional adaptations or species. The restriction of response to specific soil horizons at different sites, and lack of consistency of response is supported by similar results or lack of significant soil change, either chemical or biological, in other studies^6,13,19,22,49^.

The site and amendment-rate dependence of the enzyme responses were apparent in the results of the Wilcoxon tests. Phosphatase results were even more inconsistent than N-acetylglucosaminidase, with significant increases in ILK treatments, but only at a different amendment level for each soil horizon and, HLB treatments had decreases in PHOS restricted to the L horizon. The ILK and HLB sites were the most responsive, but they had different responses. The ILK and HLB sites varied in quite a few aspects, particularly forest structure and harvesting intensity, which could be driving the different responses. These sites also had very different litter compositions, with HLB featuring deciduous leaves and understory plants, while ILK had pine needles and any mosses that survived harvesting. It may be that sites that are more established respond to ash amendment in ways that decrease metabolic activity (e.g., pH change outside of optimum ranges for enzyme activity), while younger stands are more likely to respond positively to the application of wood ash. This explanation supports why ALS and HLB sites had reduced enzymatic activities due to ash treatment, while the ALN, ETM, ILK, PLD and SRD sites had positive responses. That the ALN and ALS sites did not respond similarly could be related to its fire history. The historical burn at the ALS site could have altered the response to ash treatment, as ash application and fire can contribute similar compounds to soil (Figs. 3, 4). Alternatively, enzyme activity responses may just signal shifts in nutrient acquisition strategy, and not net reductions in activity. In an earlier study, Noyce et al.^19^ did not observe changes in microbial respiration at soils from the ILK or HLB sites relatively shortly after ash amendment. Pugliese et al.^49^ also did not find that ash addition at a rate of 5 Mg ha^−1^ to HLB soils had an effect on respiration. If this pattern held true in 2017, then the decreases in enzyme activity at the HLB site may reflect a higher availability of nitrogen and phosphorus, decreasing the need for the production of enzymes required to scavenge these nutrients from organic materials. Bang-Andreasen et al.^50^ show that there are up-regulations in microbial growth associated genes with higher levels of ash amendment (12 Mg ha^−1^, 90 Mg ha^−1^), but no response at a lower amendment rate (3 Mg ha^−1^). In a following study, Paredes et al.^61^ found that as low as 9 Mg ha^−1^ could alter bacterial communities, without changing fungal community structure. Our amendment rates may be large enough to modify the metabolic strategies of organisms, but not so large as to affect growth or provide selective pressures for particular functional attributes in systems with less severe disturbance. Ash addition at the amendment rates in this study may provide benefits to forest ecosystems that are freshly disturbed, by being incorporated into initial disturbance events and aiding recovery, but have more subtle effects on the microbial activity of more established forest systems where there are less nutrient limitation, low water holding capacity and acidity stressors.

The measured responses to ash and site characteristics were variable. The inconsistency of response affected the ability to model enzyme activity responses to ash addition. Modeling of effects across sites was inconclusive as the models appeared to be driven by activity differences at few sites. The variance that was explained did suggest that changes in MIN soil horizons were driven more by indirect climate and site characteristics and that changes to enzyme activities within ORG horizons were driven more directly by soil chemical characteristics and ash chemistry (Fig. 2). It may be that functional responses are temporary and may transition through the soil profile over time. Gomoyrova et al.^22^ observed that functional responses were limited to organic horizons and progressed through those horizons during a six month trial. Sites in this study showed both increased and decreased activities in response to ash amendment in both layers even when the application was within two years of the sampling. It should be noted though, that the different soil layers that did have response could be because of the movement of the effect through the soil profile over time. Many studies on microbial activity have been short-lived^19,49^. The time-frame where ash amendment is most influential has yet to be established, with suggestions that effects may appear in mineral horizons within the first few months after application or not until years after application^6,22,62,63^. Stand type, site history, soil characteristics and climate variables are understandably influential on the responses of PHOS and NAG activities to ash amendment. Tree composition can also have an influence on the mineral soil community, through direct effects in root associations, or through indirect effects via their effects on litter composition and understory vegetation^43^. Climate and soil variables are equally important, as they influence soil moisture, chemistry, and, temperature. Previous studies have also shown that soil type is an important consideration for wood ash amendment^65^. Soil moisture, chemistry and temperature can be limiting on microbial community composition, growth and activity, as well as contribute to weathering and leaching of ash materials^9,66^. Though the mechanisms that drove the few observed responses in enzyme activity cannot be determined with surety, these results demonstrate the importance of considering site characteristics and climate regimes when evaluating the effects of wood ash amendment.

Metabarcoding community responses were also generally site-specific or nonexistent. Differences in community structure between ash treatments and controls were comparable to the differences between controls within sites, as evidenced by the small amount of compositional variance explained by ash related parameters and, the control-treatment distances that were comparable or lower than control-control distances (Supplemental Materials Figs. S2, S3). The limited number of organisms found to have potential increases or decreases after amendment were all eukaryotic and no single type of organism (parasite, rhizosphere-associate) universally benefited or was negatively affected across the study sites. Only three site-treatment combinations had a group that was universally lost or gained from the site after treatment (SRD 10 Mg ha^−1^, ALN CPLP Ash 5 Mg ha^−1^, ALS-A2 CPLP Ash 5 Mg ha^−1^). As eukaryotic DNA signatures are more susceptible to random sampling biases due to their multicellular nature, and varied sizes (i.e., cells are attached, and less likely to be evenly distributed in samples), this is not particularly strong evidence of community shift. As there was also little evidence of changes to soil quality or pH in these sites, the lack of community response to ash addition is likely because the changes to soil characteristics were minor to non-detectable^18^. It has been demonstrated that harvesting can cause detectable community changes^17^. While the quantities of wood ash added in this study are considered reasonable operational amount, they might have been insufficient to mitigate stresses (e.g., calcium and magnesium depletion, high acidity) and create further community shifts. Our highest amendment rate of 20 Mg ha^−1^ was much lower than the 90 Mg ha^−1^ required to observe changes to bacterial and fungal community composition in Bang-Andreasen et al.^50^ There is another potential explanation for the lack of observed changes; there has been some evidence that the high calcium content and pH ameliorating effects of ash mitigates toxicity of metal components^53,60^. It may be that competing interactions of ash components negate each other, or prevent use of those components, buffering any positive or negative effects of the wood ash amendments.

## Conclusion

The interactions between site characteristics, ash characteristics and ash application rates remain complex. Soil communities did not have large responses to ash amendment, and there were few sites and treatments which resulted in truly disruptive changes, (i.e., changes found in multiple metabarcoding targets). Though we did see soil enzyme activity responses, they were limited and inconsistent. The enzyme and community responses that did occur were not correlated, i.e., there were not consistent community shifts that corresponded to enzyme responses, indicating short-term functional responses, rather than long-term changes. While our results allowed us to determine that site characteristics are important in determining responses to ash amendment, our study was limited in its capacity to determine what drives these interactions. Finally, there was no strong response to the constituents added via ash amendment, showing that ash properties alone are not good predictors of response. Though there do not appear to be any consistently beneficial or deleterious effects to soil function or community structure, the potential benefits (e.g., increased tree growth) of ash amendment are apparent. There are many studies that indicate positive effects of ash amendment, and risks of heavy metal toxicity appear to be minimal. Current guidelines and sufficient economic incentives may justify the use of ash as an amendment. There is interest in applying ash in commercial harvesting operations, but also in small-scale private land-management^13^. It may be worth investigating the time-frame and harvesting practices in which ash should be applied, as there may be different functional effects when ash is applied to older or less intensive harvesting systems compared to clearcut systems where regeneration is at an early stage. The number of applications should also be investigated, our study only looked at single applications, but it may be that different application strategies result in different effects. It may also be that ash is more effective at sites without N limitation, further studies should evaluate whether N-limitation influences the effectiveness of ash. This study did not find strong effects of ash amendment on arthropod, bacterial and fungal community composition, and further studies should expect small, inconsistent effects that may require highly intensive sampling and replication to detect.

## Supplementary Information

Supplementary Information.

## Data Availability

All summarized data used in this experiment and supplemental materials results are available on the public github repository located at https://github.com/smendero/AshNet_Biodiversity, de-multiplexed Illumina paired-end reads are available through the NCBI SRA Bioproject PRJNA751922. Infiles and scripts are available from the author's github page https://github.com/smendero/AshNet_Biodiversity. MetaWorks is available from https://github.com/terrimporter/MetaWorks. The reference set used with the COI classifier is available from https://github.com/terrimporter/CO1Classifier, the 18S classifier is available from https://github.com/terrimporter/18SClassifier, and the ITS classifier is available from https://github.com/terrimporter/UNITE_ITSClassifier.
