# Supplementary material for: Forest soil biotic communities show few responses to wood ash applications at multiple sites across Canada
Source: Sci Rep. 2022 Mar 9;12:4171. doi: 10.1038/s41598-022-07670-x (PMC8907164; doi:10.1038/s41598-022-07670-x)
Supplement: Supplementary file 1 — Supplementary Information. [file 41598_2022_7670_MOESM1_ESM.html]

Supplemental Materials


# Supplemental Materials

#### Emily Smenderovac

#### 2021-11-23

# Methods

## Metabarcoding analysis

DNA from all samples were extracted using the Qiagen DNEasy Power Soil kit. Amplification of 18S, CO1-F230 and, ITS sequences were performed at the Great Lakes Forestry Centre, Sault Ste. Marie using the primer sets in (Table S1). Triplicate PCR reactions were performed on each sample using primers using HotStarTaq Plus with illumina adaptor sequences, pooled, purified and quantified using the QiaCube fluorometric quantification. PCR reaction conditions are listed in Table S2.
Metabarcoding was performed on the Illumina MiSeq platform at the Centre for Biodiversity Genomics, University of Guelph for 18S, CO1-F230 and, ITS amplicons. Amplification of amplicons and Metabarcoding were performed at Metagenombio for 16S amplicons from submitted environmental DNA.

Table S1:  Base primers used for sequence amplification.


|  | target | forward | reverse |
| --- | --- | --- | --- |
| d16S | 16S v4-v5 | 5’-CCTACGGGNBGCASCAG1 | 5’-GACTACNVGGGTATCTAATCC1 |
| d18S | 18S v4 | 5’- CCAGCASCYGCGGTAATTCC2 | 5’- ACTTTCGTTCTTGATYRA2 |
| CO1F230 | CO1 - F230 | 5’- GGTCAACAAATCATAAAGATATTGG3 | 5’- CTTATRTTRTTTATICGIGGRAAIGC4 |
| ITS2 | ITS2 | 5’- GAACGCAGCRAAIIGYGA5 | 5’- TCCTCCGCTTATTGATATGC6 |

In addition to the main primer sequence, primers used for amplification of 18S, ITS, and COI sequences had an illumina p5 adapter (5’-TCGTCGGCAGCGTCAGATGTGTATAAGAGACAG-3’) fused to the 5’ end of the forward primer, and another illumina p7 adapter (5’ -GTCTCGTGGGCTCGGAGATGTGTATAAGAGACAG-primer-3’) fused to the 5’ end of the reverse primer.

Table S2:  PCR conditions used for sequence amplifications performed at the Great Lakes Forestry Centre, Sault Ste. Marie.


|  | target | PCR conditions |
| --- | --- | --- |
| d18S | 18S v4 | 95°C for 5 min, 5 cycles (94°C for 45 s, 54°C for 45 s, 72°C for 45 s), 25 cycles (94°C for 45 s, 47°C for 45 s, 72°C for 45 s), 72°C for 10 min |
| CO1F230 | CO1 - F230 | 95°C for 5 min, 30 cycles (94°C for 45 s, 43°C for 45 s, 72°C for 45 s), 72°C for 10 min |
| ITS2 | ITS2 | 95°C for 5 min, 30 cycles (94°C for 45 s, 53°C for 45 s, 72°C for 45 s), 72°C for 10 min |

Table S3:  Summary statistics from read merging and primer trimming via the MetaWorks v1.4.0 pipeline


| Step | Total seq number (across samples) | Mean seq number (per sample) | min seq length | max seq length | mean seq length | amplicon |
| --- | --- | --- | --- | --- | --- | --- |
| R1 | 8028118 | 46948.06 | 251.00000 | 251.0000 | 251.0000 | 16S |
| R2 | 8028118 | 46948.06 | 251.00000 | 251.0000 | 251.0000 | 16S |
| paired | 5898010 | 34491.29 | 79.57895 | 455.9942 | 411.7317 | 16S |
| Ftrimmed | 5781281 | 33808.66 | 314.18713 | 436.1988 | 392.7466 | 16S |
| Rtrimmed | 5776828 | 33782.62 | 319.52047 | 416.0526 | 372.8795 | 16S |
| R1 | 85517092 | 186718.54 | 35.55895 | 301.0000 | 295.9288 | 18S |
| R2 | 85517092 | 186718.54 | 35.53275 | 301.0000 | 296.1581 | 18S |
| paired | 76288757 | 166569.34 | 36.15502 | 486.7183 | 355.7121 | 18S |
| Ftrimmed | 19706028 | 43026.26 | 173.80568 | 466.3253 | 398.7665 | 18S |
| Rtrimmed | 19571897 | 42733.40 | 179.22926 | 448.3777 | 380.9189 | 18S |
| R1 | 85517092 | 186718.54 | 35.55895 | 301.0000 | 295.9288 | F230 |
| R2 | 85517092 | 186718.54 | 35.53275 | 301.0000 | 296.1581 | F230 |
| paired | 76288757 | 166569.34 | 36.15502 | 486.7183 | 355.7121 | F230 |
| Ftrimmed | 17889517 | 39060.08 | 184.94541 | 374.3799 | 258.3421 | F230 |
| Rtrimmed | 17856519 | 38988.03 | 181.49345 | 342.3668 | 232.4368 | F230 |
| R1 | 85517092 | 186718.54 | 35.55895 | 301.0000 | 295.9288 | ITS |
| R2 | 85517092 | 186718.54 | 35.53275 | 301.0000 | 296.1581 | ITS |
| paired | 76288757 | 166569.34 | 36.15502 | 486.7183 | 355.7121 | ITS |
| Ftrimmed | 25350300 | 55350.00 | 216.76856 | 466.3712 | 345.0956 | ITS |
| Rtrimmed | 25285054 | 55207.54 | 207.23144 | 446.3712 | 325.1651 | ITS |

Table S4:  Summary statistics from dereplication and chimera filtering via the MetaWorksv1.4.0 pipeline


| Step | number | min | max | mean | amplicon |
| --- | --- | --- | --- | --- | --- |
| dereplication | 2984905 | 167 | 438 | 373 | 16S |
| chimera removal | 16S/cat.denoised: 22917/75358 chimeras (30.4%) | 339 | 425 | 373 | 16S |
| dereplication | 8296788 | 150 | 458 | 381 | 18S |
| chimera removal | 18S/cat.denoised: 11306/42971 chimeras (26.3%) | 150 | 449 | 380 | 18S |
| dereplication | 3172677 | 150 | 436 | 232 | F230 |
| chimera removal | F230/cat.denoised: 2900/35935 chimeras (8.1%) | 150 | 430 | 233 | F230 |
| dereplication | 8955577 | 150 | 454 | 325 | ITS |
| chimera removal | ITS/cat.denoised: 5852/34438 chimeras (17.0%) | 152 | 450 | 320 | ITS |

The rarecurve function in vegan was used to visually examine samples for sufficient read depths (whether the number of ASVs reached a plateau) before it was decided that data analysis could proceed without rarefying7.

Table S5: Percentage of ASV classified to Genus or assigned to a functional attribute for each metabarcoding target.

| Target | Number of ASV | Percent of ASV identified to Genus | Percent of ASV assigned to a functional attribute |
| --- | --- | --- | --- |
| ITS | 22083 | 49.50414 | 64.03744 |
| 16S | 27539 | 65.68140 | 48.78898 |
| F230 | 7923 | 100.00000 | 39.24602 |
| 18S | 22166 | 23.18867 | NA |

## Statistical testing

Table S6:  Summary of statistical tests run in this paper.

| Response variable | Test | Explanatory variables | Random variables | Data subsets |
| --- | --- | --- | --- | --- |
| Enzyme activity | Mixed-effects ANOVA | ash addition, ash addition amount | site, soil type | Full datasets for NAG and PHOS activities |
| Enzyme activity | Fixed-effects ANOVA | ash addition, ash addition amount, site, soil type with interaction | None | Full datasets for PHOS and NAG activities |
| Enzyme activity | lm | applied calcium kg ha-1, applied phosphorus kg ha-1, and, applied sodium kg ha-1, Stand age, dominant tree species, precipitaion of wettest quarter, precipitation of seasonality | None | Full datasets for PHOS and NAG activities |
| Difference in enzyme activity between controls and treatment for each block | One-way Wilcoxon test | None | None | Each site, ash type and amendment rate evaluated individually for NAG and PHOS |
| One model for each diversity metric (Shannon, Inverse Simpsons, Richness) | Mixed-effects ANOVA | ash addition, ash addition amount | site, soil type | Full dataset |
| Difference between controls and treatment for each block model for each diversity metric (Shannon, Inverse Simpsons, Richness) | One-way Wilcoxon test | None | None | Each site, ash type and amendment rate evaluated individually |
| Compositional variance of each metabarcoding target and summarization level | PCA | None | None | Full dataset |
| Compositional variance of each metabarcoding target and summarization level | Partial RDA | applied calcium kg ha-1, applied phosphorus kg ha-1, and, applied sodium kg ha-1 | Site, soil type |  |
| Compositional variance of each group for each metabarcoding target and summarization level | Aldex glm | applied calcium kg ha-1, applied phosphorus kg ha-1, and, applied sodium kg ha-1, Stand age, dominant tree species, precipitaion of wettest quarter, precipitation of seasonality | None | Soil type |
| Difference between control and treatment compositional variance of each group for each metabarcoding target and summarization level | Aldex pairwise analysis | None | None | Each site, ash type and amendment rate evaluated individually |

# Results

## Enzyme Analyses

Table S7:  Type III mixed-effects Analysis of variance results for phosphatase activity as explained by ash amendment (Ash) and amount of ash amendment (ash\_amt) after site and soil horizon (soil\_type) were accounted for as random effects.

| Parameter | Chisq | Df | Pr(>Chisq) |
| --- | --- | --- | --- |
| (Intercept) | 8.8314388 | 1 | 0.003 |
| Ash | 0.7055555 | 1 | 0.401 |
| ash\_amt | 0.5735164 | 1 | 0.449 |

Table S8:  Type III fixed-effects Analysis of variance results for phosphatase activity as explained by site, soil horizon (soil\_type), ash amendment (Ash) and amount of ash amendment (ash\_amt).

| Parameter | Sum Sq | Df | F values | Pr(>F) |
| --- | --- | --- | --- | --- |
| Site | 550,409.479 | 7 | 0.256681098 | 0.970 |
| Soil\_type | 1,559.923 | 1 | 0.005092244 | 0.943 |
| Ash | 18,673.569 | 1 | 0.060958372 | 0.805 |
| ash\_amt | 224,654.887 | 1 | 0.733367897 | 0.392 |
| Site:Soil\_type | 5,739,893.844 | 8 | 2.342177999 | 0.019 |
| Site:Ash | 272,915.415 | 7 | 0.127272932 | 0.996 |
| Soil\_type:Ash | 53,159.777 | 1 | 0.173535835 | 0.677 |
| Site:Soil\_type:Ash | 406,188.716 | 8 | 0.165746319 | 0.995 |
| Residuals | 92,512,606.955 | 302 |  |  |

Table S9:  Type III mixed-effects Analysis of variance results for N-acetylglucosaminidase activity as explained by ash amendment (Ash) and amount of ash amendment (ash\_amt) after site and soil horizon (soil\_type) were accounted for as random effects.

| Parameter | Chisq | Df | Pr(>Chisq) |
| --- | --- | --- | --- |
| (Intercept) | 7.70105436 | 1 | 0.006 |
| Ash | 0.41105235 | 1 | 0.521 |
| ash\_amt | 0.06724982 | 1 | 0.795 |

Table S10:  Type III fixed-effects Analysis of variance results for N-acetylglucosaminidase activity as explained by site, soil horizon (soil\_type), ash amendment (Ash) and amount of ash amendment (ash\_amt).

| Parameter | Sum Sq | Df | F values | Pr(>F) |
| --- | --- | --- | --- | --- |
| Site | 30,061.565 | 7 | 0.04401825 | 1.000 |
| Soil\_type | 250,582.516 | 1 | 2.56844307 | 0.110 |
| Ash | 3,906.410 | 1 | 0.04004027 | 0.842 |
| ash\_amt | 10,525.786 | 1 | 0.10788814 | 0.743 |
| Site:Soil\_type | 22,232,144.065 | 8 | 28.48462718 | 0.000 |
| Site:Ash | 20,997.642 | 7 | 0.03074622 | 1.000 |
| Soil\_type:Ash | 2,522.354 | 1 | 0.02585385 | 0.872 |
| Site:Soil\_type:Ash | 8,854,943.389 | 8 | 11.34527378 | 0.000 |
| Residuals | 29,463,732.606 | 302 |  |  |

## Community Analyses

### Alpha Diversity

Table S11: Type III Analysis of variance results for diversity metrics for each metabarcoding target as explained by ash amendment (Ash) and amount of ash amendment (ash\_amt) after accounting for effects of site and soil horizon (soil type).

| Amplicon | Diversity metric | Parameter | Chisq | Degrees of Freedom | Pr(>Chisq) |
| --- | --- | --- | --- | --- | --- |
| ITS | Shannon | (Intercept) | 1,359.926 | 1 | 0.000 |
| ITS | Shannon | Ash | 0.015 | 1 | 0.901 |
| ITS | Shannon | ash\_amt | 0.971 | 1 | 0.324 |
| ITS | InverseSimpson | (Intercept) | 57.045 | 1 | 0.000 |
| ITS | InverseSimpson | Ash | 0.191 | 1 | 0.662 |
| ITS | InverseSimpson | ash\_amt | 1.473 | 1 | 0.225 |
| ITS | richness | (Intercept) | 57.045 | 1 | 0.000 |
| ITS | richness | Ash | 0.191 | 1 | 0.662 |
| ITS | richness | ash\_amt | 1.473 | 1 | 0.225 |
| 16S | Shannon | (Intercept) | 1,786.247 | 1 | 0.000 |
| 16S | Shannon | Ash | 1.434 | 1 | 0.231 |
| 16S | Shannon | ash\_amt | 0.499 | 1 | 0.480 |
| 16S | InverseSimpson | (Intercept) | 32.931 | 1 | 0.000 |
| 16S | InverseSimpson | Ash | 0.337 | 1 | 0.561 |
| 16S | InverseSimpson | ash\_amt | 2.742 | 1 | 0.098 |
| 16S | richness | (Intercept) | 125.379 | 1 | 0.000 |
| 16S | richness | Ash | 0.035 | 1 | 0.852 |
| 16S | richness | ash\_amt | 0.000 | 1 | 0.989 |
| F230 | Shannon | (Intercept) | 195.349 | 1 | 0.000 |
| F230 | Shannon | Ash | 0.891 | 1 | 0.345 |
| F230 | Shannon | ash\_amt | 1.583 | 1 | 0.208 |
| F230 | InverseSimpson | (Intercept) | 15.204 | 1 | 0.000 |
| F230 | InverseSimpson | Ash | 0.101 | 1 | 0.751 |
| F230 | InverseSimpson | ash\_amt | 0.010 | 1 | 0.920 |
| F230 | richness | (Intercept) | 15.204 | 1 | 0.000 |
| F230 | richness | Ash | 0.101 | 1 | 0.751 |
| F230 | richness | ash\_amt | 0.010 | 1 | 0.920 |
| 18S | Shannon | (Intercept) | 1,509.821 | 1 | 0.000 |
| 18S | Shannon | Ash | 1.329 | 1 | 0.249 |
| 18S | Shannon | ash\_amt | 0.617 | 1 | 0.432 |
| 18S | InverseSimpson | (Intercept) | 62.995 | 1 | 0.000 |
| 18S | InverseSimpson | Ash | 1.008 | 1 | 0.315 |
| 18S | InverseSimpson | ash\_amt | 0.733 | 1 | 0.392 |
| 18S | richness | (Intercept) | 62.995 | 1 | 0.000 |
| 18S | richness | Ash | 1.008 | 1 | 0.315 |
| 18S | richness | ash\_amt | 0.733 | 1 | 0.392 |

Figure S1: Scaled differences in alpha diversity metrics between treatments and controls of blocks within each site.

Table S12: Table 1. Distribution of the alpha diversity pairwise wilcoxon test results for site and treatment combinations with at least one significant result. Tests for ASV, genus and functional groups are represented by A, G and F respectively and bolded where the result was significant at a Bonferonni corrected \(\alpha\) of 0.05. 92 of 1494 tests are displayed in this table with only 28 significant results (~2%).

| Site | Soil Type | diversity\_metric | Mg/ha Ash | Type of Ash | 18S | F230 | ITS | 16S |
| --- | --- | --- | --- | --- | --- | --- | --- | --- |
| HLB | 0-10 cm mineral soil | InverseSimpson | 4.0 | Bottom | **A** **G** | A F G | A F G | NA |
| HLB | 0-10 cm mineral soil | richness | 4.0 | Bottom | **A** **G** | A F G | A F G | NA |
| HLB | 0-10 cm mineral soil | Shannon | 4.0 | Bottom | **A** **G** | A F G | A F G | NA |
| ILK | 0-10 cm mineral soil | InverseSimpson | 0.7 | Bottom | A G | A F **G** | A F G | A F G |
| ILK | 0-10 cm mineral soil | InverseSimpson | 2.8 | Bottom | A G | **A** F G | A F G | A F G |
| ILK | 0-10 cm mineral soil | InverseSimpson | 5.6 | Bottom | A G | A F G | **A** F G | A F G |
| ILK | 0-10 cm mineral soil | richness | 0.7 | Bottom | A G | A F **G** | A F G | A F G |
| ILK | 0-10 cm mineral soil | richness | 2.8 | Bottom | A G | **A** F G | A F G | A F G |
| ILK | 0-10 cm mineral soil | richness | 5.6 | Bottom | A G | A F G | **A** F G | A F G |
| ILK | 0-10 cm mineral soil | Shannon | 0.7 | Bottom | A G | A F **G** | A F G | A F G |
| ILK | 0-10 cm mineral soil | Shannon | 2.8 | Bottom | A G | **A** F G | A F G | A F G |
| HLB | FH-layer forest floor | InverseSimpson | 4.0 | Bottom | A G | A **F** G | A F G | NA |
| HLB | FH-layer forest floor | richness | 4.0 | Bottom | A G | A **F** G | A F G | NA |
| HLB | FH-layer forest floor | Shannon | 4.0 | Bottom | A G | A **F** G | A F G | NA |
| HLB | FH-layer forest floor | Shannon | 8.0 | Fly | A G | A **F** G | A F G | A F G |
| ETM | surface litter &/or moss | InverseSimpson | 20.0 | Bottom | A G | A F **G** | A F G | NA |
| ETM | surface litter &/or moss | richness | 20.0 | Bottom | A G | A F **G** | A F G | NA |
| HLB | surface litter &/or moss | InverseSimpson | 1.0 | Bottom | A G | A F **G** | A F G | NA |
| HLB | surface litter &/or moss | InverseSimpson | 4.0 | Bottom | A G | A F **G** | A F G | NA |
| HLB | surface litter &/or moss | InverseSimpson | 4.0 | Fly | A G | A F **G** | A F G | A F G |
| HLB | surface litter &/or moss | InverseSimpson | 8.0 | Bottom | A G | A F **G** | A F G | NA |
| HLB | surface litter &/or moss | richness | 1.0 | Bottom | A G | A F **G** | A F G | NA |
| HLB | surface litter &/or moss | richness | 4.0 | Bottom | A G | A F **G** | A F G | NA |
| HLB | surface litter &/or moss | richness | 4.0 | Fly | A G | A F **G** | A F G | A F G |
| HLB | surface litter &/or moss | richness | 8.0 | Bottom | A G | A F **G** | A F G | NA |
| HLB | surface litter &/or moss | Shannon | 1.0 | Bottom | A G | A F **G** | A F G | NA |
| HLB | surface litter &/or moss | Shannon | 4.0 | Bottom | A G | A F **G** | A F G | NA |
| HLB | surface litter &/or moss | Shannon | 4.0 | Fly | A G | A F **G** | A F G | A F G |
| HLB | surface litter &/or moss | Shannon | 8.0 | Bottom | A G | A F **G** | A F G | NA |

Island Lake was the only site where the distances between ash-treatment and controls were larger than the differences between control replicates within plots. Though there were higher distances at Genus and Functional levels as well, only ASV is used is discussion due to the interpretation issues that are introduced from incomplete Genus and functional assignments.

Figure S2: Scaled difference in ASV community distance (Jaccard for Presence-Absence or Bray-Curtis for rarefied data) between Ash Addition plots compared to controls to distance between controls for each block. Only Island Lake is shown, as no other sites had any distances that were significantly higher than controls at \(\alpha\) = 0.05.

### CODA PCA

Figure S3: Compositional variance explained in first two PC axes for each metabarcoding dataset.

### CODA RDA

Figure S4: RDA of ITS ASV groups. Control sites are shown as + symbols with a solid line surrounding their distribution, while samples with ash amendment are shown as transparent circles sized to reflect the amount of ash added and surrounding with a transparent hull showing their distribution. Colour is used to represent the soil source of the sample. Arrows represent ash additions

Figure S5: RDA of 16S ASV groups. Control sites are shown as + symbols with a solid line surrounding their distribution, while samples with ash amendment are shown as transparent circles sized to reflect the amount of ash added and surrounding with a transparent hull showing their distribution. Colour is used to represent the soil source of the sample. Arrows represent ash additions

Figure S6: RDA of F230 ASV groups. Control sites are shown as + symbols with a solid line surrounding their distribution, while samples with ash amendment are shown as transparent circles sized to reflect the amount of ash added and surrounding with a transparent hull showing their distribution. Colour is used to represent the soil source of the sample. Arrows represent ash additions

Figure S7: RDA of 18S ASV groups. Control sites are shown as + symbols with a solid line surrounding their distribution, while samples with ash amendment are shown as transparent circles sized to reflect the amount of ash added and surrounding with a transparent hull showing their distribution. Colour is used to represent the soil source of the sample. Arrows represent ash additions

### Modeling of distinct species

```
## Warning: Vectorized input to `element_text()` is not officially supported.
## Results may be unexpected or may change in future versions of ggplot2.
```

Figure S8: Benjamini-Hochberg corrected p-values from aldex-glms performed on ASV, Genus level and functional tables from 16S, 18S, ITS and F230 datasets. \(\alpha\) = 0.05 is shown as a lightly coloured dashed line. Parameters associated with ash quality are bolded.

Ash phosphorus addition was a significantly associated with changes in the compositonal amount of some arthropod ASVs. There were no additional significant associations of ash related paramenters to the centered log-ratio values for any targeted group after Benjamini-Hochberg correction was performed.

When assessed via pairwise comparisons using compositional t-tests, controls and treatments did not have any ASVs, genus or functional groups that were identified as significantly different (\(\alpha\) = 0.05).

```
## Warning: Vectorized input to `element_text()` is not officially supported.
## Results may be unexpected or may change in future versions of ggplot2.
```

Figure S9: Benjamini-Hochberg corrected p-values from glms on the gain or loss of a target group as compared to a control site. Glms were performed on ASV, Genus level and functional tables from 16S, 18S, ITS and F230 datasets. \(\alpha\) = 0.05 is shown as a lightly coloured dashed line. Parameters associated with ash quality are bolded.

Figure S10: Proportion of sites with changes in gain/loss of a target group significantly (B-H p <= 0.05) associated with an ash-amendment related parameter.

Upon visual inspection of the percentage of sites that had a gain or loss, patterns in ASV or genus that were found to be significantly associated to estimated total phosphorus or total calcium in the applied ash were site dependent, or showed conflicting patterns in different sites (e.g., Losses of *Uroleptus* in SRD and Gains at ALN, ALS sites)

## Traditional diversity analysis

We also ran some more traditional NMDS and beta-diversity analyses using the vegan package in R, which found a lack of consistent influence of ash additions on community composition. Bacterial (16S) datasets were assessed as relative abundance using Bray-Curtis distance, and all other datasets were assessed as presence/absence matrixes using Jaccard distances..

Community assemblages were visually different for some sites at the ASV level. These differences were not consistently present when datasets were summarized at genus or functional levels, which can be at least partially attributed to the loss of ASVs that could not be identified at these levels (Supplemental Results: Figures S12 - S22).

Figure S11: Ordination of ITS ASV groups. Control sites are shown as + symbols with a solid line surrounding their distribution, while samples with ash amendment are shown as transparent circles sized to reflect the amount of ash added and surrounding with a transparent hull showing their distribution. Colour is used to represent the soil source of the sample. There was noticeable overlap between treatments and controls in most sites, for most soil layers.

Figure S12: Ordination of ITS functional groups. Control sites are shown as + symbols with a solid line surrounding their distribution, while samples with ash amendment are shown as transparent circles sized to reflect the amount of ash added and surrounding with a transparent hull showing their distribution. Colour is used to represent the soil source of the sample. There was noticeable overlap between treatments and controls in most sites, for most soil layers.

Figure S13: Ordination of ITS Genus groups. Control sites are shown as + symbols with a solid line surrounding their distribution, while samples with ash amendment are shown as transparent circles sized to reflect the amount of ash added and surrounding with a transparent hull showing their distribution. Colour is used to represent the soil source of the sample. There was noticeable overlap between treatments and controls in most sites, for most soil layers.

Figure S14: Ordination of 16S ASV groups. Control sites are shown as + symbols with a solid line surrounding their distribution, while samples with ash amendment are shown as transparent circles sized to reflect the amount of ash added and surrounding with a transparent hull showing their distribution. Colour is used to represent the soil source of the sample. There was noticeable overlap between treatments and controls in most sites, for most soil layers.

Figure S15: Ordination of 16S functional groups. Control sites are shown as + symbols with a solid line surrounding their distribution, while samples with ash amendment are shown as transparent circles sized to reflect the amount of ash added and surrounding with a transparent hull showing their distribution. Colour is used to represent the soil source of the sample. There was noticeable overlap between treatments and controls in most sites, for most soil layers.

Figure S16: Ordination of 16S Genus groups. Control sites are shown as + symbols with a solid line surrounding their distribution, while samples with ash amendment are shown as transparent circles sized to reflect the amount of ash added and surrounding with a transparent hull showing their distribution. Colour is used to represent the soil source of the sample. There was noticeable overlap between treatments and controls in most sites, for most soil layers.

Figure S17: Ordination of F230 ASV groups. Control sites are shown as + symbols with a solid line surrounding their distribution, while samples with ash amendment are shown as transparent circles sized to reflect the amount of ash added and surrounding with a transparent hull showing their distribution. Colour is used to represent the soil source of the sample. There was noticeable overlap between treatments and controls in most sites, for most soil layers.

Figure S18: Ordination of F230 functional groups. Control sites are shown as + symbols with a solid line surrounding their distribution, while samples with ash amendment are shown as transparent circles sized to reflect the amount of ash added and surrounding with a transparent hull showing their distribution. Colour is used to represent the soil source of the sample. There was noticeable overlap between treatments and controls in most sites, for most soil layers.

Figure S19: Ordination of F230 Genus groups. Control sites are shown as + symbols with a solid line surrounding their distribution, while samples with ash amendment are shown as transparent circles sized to reflect the amount of ash added and surrounding with a transparent hull showing their distribution. Colour is used to represent the soil source of the sample. There was noticeable overlap between treatments and controls in most sites, for most soil layers.

Figure S20: Ordination of Eukaryote ASV groups. Control sites are shown as + symbols with a solid line surrounding their distribution, while samples with ash amendment are shown as transparent circles sized to reflect the amount of ash added and surrounding with a transparent hull showing their distribution. Colour is used to represent the soil source of the sample. There was noticeable overlap between treatments and controls in most sites, for most soil layers.

Figure S21: Ordination of Eukaryote Genus groups. Control sites are shown as + symbols with a solid line surrounding their distribution, while samples with ash amendment are shown as transparent circles sized to reflect the amount of ash added and surrounding with a transparent hull showing their distribution. Colour is used to represent the soil source of the sample. There was noticeable overlap between treatments and controls in most sites, for most soil layers.

Table S13: Results of mixed effects adonis testing of different metabarcode targets. Ash and ash amount (ash\_amt) as well as site and soil horizon (Soil\_type) were included in the models.

| Dataset | Parameter | Degrees of Freedom | Sums of Squares | MeanSqs | F.Model | R2 | Pr(>F) |
| --- | --- | --- | --- | --- | --- | --- | --- |
| ITS\_ASV | Site | 7 | 33.9741707 | 4.8534530 | 13.7167935 | 0.1668752 | 0.001 |
| ITS\_ASV | Soil\_type | 3 | 13.2225858 | 4.4075286 | 12.4565253 | 0.0649470 | 0.001 |
| ITS\_ASV | Ash | 1 | 0.5289887 | 0.5289887 | 1.4950240 | 0.0025983 | 0.013 |
| ITS\_ASV | ash\_amt | 1 | 0.5319151 | 0.5319151 | 1.5032945 | 0.0026127 | 0.020 |
| ITS\_ASV | Residuals | 439 | 155.3326473 | 0.3538329 | NA | 0.7629668 | NA |
| ITS\_ASV | Total | 451 | 203.5903076 | NA | NA | 1.0000000 | NA |
| ITS\_functional | Site | 7 | 0.2233970 | 0.0319139 | 2.0801489 | 0.0313354 | 0.001 |
| ITS\_functional | Soil\_type | 3 | 0.1335244 | 0.0445081 | 2.9010442 | 0.0187291 | 0.001 |
| ITS\_functional | Ash | 1 | 0.0259919 | 0.0259919 | 1.6941578 | 0.0036458 | 0.140 |
| ITS\_functional | ash\_amt | 1 | 0.0111311 | 0.0111311 | 0.7255246 | 0.0015613 | 0.601 |
| ITS\_functional | Residuals | 439 | 6.7351830 | 0.0153421 | NA | 0.9447283 | NA |
| ITS\_functional | Total | 451 | 7.1292274 | NA | NA | 1.0000000 | NA |
| ITS\_Genus | Site | 7 | 28.0613012 | 4.0087573 | 22.4071059 | 0.2279515 | 0.001 |
| ITS\_Genus | Soil\_type | 3 | 15.9488643 | 5.3162881 | 29.7156004 | 0.1295581 | 0.001 |
| ITS\_Genus | Ash | 1 | 0.2728413 | 0.2728413 | 1.5250571 | 0.0022164 | 0.054 |
| ITS\_Genus | ash\_amt | 1 | 0.2794783 | 0.2794783 | 1.5621548 | 0.0022703 | 0.058 |
| ITS\_Genus | Residuals | 439 | 78.5395699 | 0.1789056 | NA | 0.6380037 | NA |
| ITS\_Genus | Total | 451 | 123.1020550 | NA | NA | 1.0000000 | NA |
| 16S\_ASV | Site | 6 | 19.6980473 | 3.2830079 | 18.4602692 | 0.3334116 | 0.001 |
| 16S\_ASV | Soil\_type | 3 | 10.8294750 | 3.6098250 | 20.2979535 | 0.1833011 | 0.001 |
| 16S\_ASV | Ash | 1 | 0.2119635 | 0.2119635 | 1.1918656 | 0.0035877 | 0.223 |
| 16S\_ASV | ash\_amt | 1 | 0.2417696 | 0.2417696 | 1.3594642 | 0.0040922 | 0.146 |
| 16S\_ASV | Residuals | 158 | 28.0990077 | 0.1778418 | NA | 0.4756074 | NA |
| 16S\_ASV | Total | 169 | 59.0802631 | NA | NA | 1.0000000 | NA |
| 16S\_functional | Site | 6 | 1.2469285 | 0.2078214 | 19.1035369 | 0.2609162 | 0.001 |
| 16S\_functional | Soil\_type | 3 | 1.7709942 | 0.5903314 | 54.2649451 | 0.3705755 | 0.001 |
| 16S\_functional | Ash | 1 | 0.0081500 | 0.0081500 | 0.7491706 | 0.0017054 | 0.490 |
| 16S\_functional | ash\_amt | 1 | 0.0341322 | 0.0341322 | 3.1375322 | 0.0071421 | 0.021 |
| 16S\_functional | Residuals | 158 | 1.7188327 | 0.0108787 | NA | 0.3596608 | NA |
| 16S\_functional | Total | 169 | 4.7790375 | NA | NA | 1.0000000 | NA |
| 16S\_Genus | Site | 6 | 10.4732088 | 1.7455348 | 30.2063369 | 0.3863799 | 0.001 |
| 16S\_Genus | Soil\_type | 3 | 7.3481268 | 2.4493756 | 42.3862441 | 0.2710887 | 0.001 |
| 16S\_Genus | Ash | 1 | 0.0751724 | 0.0751724 | 1.3008516 | 0.0027733 | 0.221 |
| 16S\_Genus | ash\_amt | 1 | 0.0791306 | 0.0791306 | 1.3693490 | 0.0029193 | 0.186 |
| 16S\_Genus | Residuals | 158 | 9.1303523 | 0.0577870 | NA | 0.3368389 | NA |
| 16S\_Genus | Total | 169 | 27.1059910 | NA | NA | 1.0000000 | NA |
| F230\_ASV | Site | 7 | 20.7381843 | 2.9625978 | 7.1325441 | 0.1090650 | 0.001 |
| F230\_ASV | Soil\_type | 3 | 6.7971089 | 2.2657030 | 5.4547487 | 0.0357469 | 0.001 |
| F230\_ASV | Ash | 1 | 0.5189532 | 0.5189532 | 1.2493955 | 0.0027292 | 0.028 |
| F230\_ASV | ash\_amt | 1 | 0.5146315 | 0.5146315 | 1.2389911 | 0.0027065 | 0.013 |
| F230\_ASV | Residuals | 389 | 161.5763621 | 0.4153634 | NA | 0.8497523 | NA |
| F230\_ASV | Total | 401 | 190.1452401 | NA | NA | 1.0000000 | NA |
| F230\_functional | Site | 7 | 6.4025259 | 0.9146466 | 7.0844409 | 0.0981975 | 0.001 |
| F230\_functional | Soil\_type | 3 | 8.3954727 | 2.7984909 | 21.6758520 | 0.1287640 | 0.001 |
| F230\_functional | Ash | 1 | 0.0714515 | 0.0714515 | 0.5534309 | 0.0010959 | 0.708 |
| F230\_functional | ash\_amt | 1 | 0.1086533 | 0.1086533 | 0.8415795 | 0.0016664 | 0.482 |
| F230\_functional | Residuals | 389 | 50.2223839 | 0.1291064 | NA | 0.7702762 | NA |
| F230\_functional | Total | 401 | 65.2004873 | NA | NA | 1.0000000 | NA |
| F230\_Genus | Site | 7 | 20.3349198 | 2.9049885 | 9.1685638 | 0.1321508 | 0.001 |
| F230\_Genus | Soil\_type | 3 | 9.5102304 | 3.1700768 | 10.0052207 | 0.0618043 | 0.001 |
| F230\_Genus | Ash | 1 | 0.3940371 | 0.3940371 | 1.2436380 | 0.0025607 | 0.104 |
| F230\_Genus | ash\_amt | 1 | 0.3857501 | 0.3857501 | 1.2174830 | 0.0025069 | 0.121 |
| F230\_Genus | Residuals | 389 | 123.2516414 | 0.3168423 | NA | 0.8009773 | NA |
| F230\_Genus | Total | 401 | 153.8765787 | NA | NA | 1.0000000 | NA |
| 18S\_ASV | Site | 7 | 23.1246266 | 3.3035181 | 9.2482505 | 0.1178996 | 0.001 |
| 18S\_ASV | Soil\_type | 3 | 15.5969925 | 5.1989975 | 14.5546747 | 0.0795204 | 0.001 |
| 18S\_ASV | Ash | 1 | 0.4903257 | 0.4903257 | 1.3726743 | 0.0024999 | 0.025 |
| 18S\_ASV | ash\_amt | 1 | 0.4706715 | 0.4706715 | 1.3176523 | 0.0023997 | 0.035 |
| 18S\_ASV | Residuals | 438 | 156.4556366 | 0.3572046 | NA | 0.7976804 | NA |
| 18S\_ASV | Total | 450 | 196.1382528 | NA | NA | 1.0000000 | NA |
| 18S\_Genus | Site | 7 | 14.1249361 | 2.0178480 | 13.9566615 | 0.1582337 | 0.001 |
| 18S\_Genus | Soil\_type | 3 | 11.4207355 | 3.8069118 | 26.3309125 | 0.1279401 | 0.001 |
| 18S\_Genus | Ash | 1 | 0.2034405 | 0.2034405 | 1.4071177 | 0.0022790 | 0.076 |
| 18S\_Genus | ash\_amt | 1 | 0.1913415 | 0.1913415 | 1.3234342 | 0.0021435 | 0.112 |
| 18S\_Genus | Residuals | 438 | 63.3258487 | 0.1445796 | NA | 0.7094037 | NA |
| 18S\_Genus | Total | 450 | 89.2663024 | NA | NA | 1.0000000 | NA |

Figure S22: Significance of pairwise PERMANOVA test results for each soil layer, site and treatment combination as compared to controls. Values lower than 0.05 were spread amongst different taxonomic levels and targets. Lower p-values indicated that there was a low probability of the community from ash treatment being the same as controls from the same site. Colour and shape are used to differentiate the targeted sequence and grouping level used for each test.

PERMANOVA tests of each metabarcode summarized at ASV, Genus and functional characteristics showed significant (p <0.05) influence of ash amendment or ash amount on community composition for ITS, F230 and 18S datasets. The majority of the variance was explained by site and soil horizon, and ash amendment explained a small proportion of the variance, with R2 values less than 0.003. Pairwise assessment of community distributions from block treatment-control pairings showed a small proportion of treatments that resulted in a shift from controls significant at \(\alpha\) = 0.05, only the ILK F230 ASV dataset FH layer was found to be significantly different between 2.8 t ha-1 wood ash amended soils and controls after using a Bonferroni-corrected alpha to account for the number of tests performed (Figure S22).

Table S14:  Distribution of the adonis results. Tests for ASV, Genus and Functional groups are represented by A, G and F respectively and bolded where the result was significant at an \(\alpha\) of 0.05.

| Site | Soil Type | Mg/ha Ash | Type of Ash | Arthropods (F230) | Eukaryotic (18S) | Fungal (ITS) | Bacterial (16S) |
| --- | --- | --- | --- | --- | --- | --- | --- |
| HLB | 0-10 cm mineral soil | 4.0 | Fly | **A** F G | A G | **A** F G |  |
| HLB | 0-10 cm mineral soil | 8.0 | Bottom | A F G | A G | A F **G** |  |
| ILK | 0-10 cm mineral soil | 1.4 | Bottom | A F G | A **G** | A F **G** | A F G |
| ILK | 0-10 cm mineral soil | 2.8 | Bottom | A F G | **A** G | A F G | A F G |
| SRD | 0-10 cm mineral soil | 10.0 | Fly | A F G | A G | **A** F G | **A** F **G** |
| HLB | FH-layer forest floor | 1.0 | Fly | **A** F G | A G | A F G | A F G |
| HLB | FH-layer forest floor | 8.0 | Bottom | A F G | A G | A **F** G |  |
| ILK | FH-layer forest floor | 0.7 | Bottom | A F **G** | A G | A F G | A F G |
| ILK | FH-layer forest floor | 1.4 | Bottom | **A** F G | **A** G | A F G | A F G |
| ILK | FH-layer forest floor | 2.8 | Bottom | **A** F **G** | **A** G | **A** F G | A F G |
| ILK | FH-layer forest floor | 5.6 | Bottom | A F G | **A** **G** | **A** F G | A F G |
| PLD | surface litter &/or moss with FH layer | 1.5 | Mixed | A F G | A G | A F **G** | A F G |
| SRD | surface litter &/or moss with FH layer | 1.0 | Fly | A **F** G | A G | A F G | A F G |

Potential differences detected in some sites occurred in both clearcut (ILK, PLD, SRD) and selection cut systems (HLB). Most of these differences were from the Island Lake site, for 18S and ITS data, indicating a fungal response. Significant (at \(\alpha\) = 0.05) PERMANOVA results did not coincide with significant differences in betadispersion, showing that these differences are likely due to a shift in average community composition, rather than a differences in community structure variance (Supplemental Results: Table S10, S11). The differences in betadispersion were mainly from the ASV level analyses of F230 (arthropod) datasets, and corresponded to NMDS analysis, suggesting that there is generally higher dispersion in the controls compared to individual treatments. The solid polygons representing treatment dispersals were generally smaller than the area encapsulated by the solid line (Supplementary Results: Figure S19, Figure S20). Only the ILK sites had shifts in community distance due to treatment that were significantly greater than distances between controls within blocks. However, these differences were comparable to the distances from between-block comparisons (Supplemental Results: Figure S2).

Figure S23: Significance of pairwise betadispersion testing from metabarcoding subsets summarized at functional, genus and ASV levels. Significance at an \(\alpha\) of 0.05 and the Bonferonni corrected \(\alpha\) for each subset are shown on the graph as a light coloured dashed line, and dark coloured solid line, respectively.

Table S15:  Distribution of betadispersion results. Tests for ASV, Genus and Functional groups are represented by A, G and F respectively and bolded where the result was significant at an \(\alpha\) of 0.05.

| Site | Soil Type | Mg/ha Ash | Type of Ash | Arthropods (F230) | Eukaryotic (18S) | Fungal (ITS) | Bacterial (16S) |
| --- | --- | --- | --- | --- | --- | --- | --- |
| ALN | 0-10 cm mineral soil | 5.0 | CPLP Bottom | **A** **F** **G** | A G | A F **G** |  |
| ALN | 0-10 cm mineral soil | 5.0 | UNBC Bottom | **A** **F** **G** | A G | A F G |  |
| ALS | 0-10 cm mineral soil | 5.0 | CPLP Bottom | **A** **F** **G** | A G | A F G |  |
| ALS | 0-10 cm mineral soil | 5.0 | UNBC Bottom | **A** F G | A G | A F G |  |
| ETM | 0-10 cm mineral soil | 20.0 | Bottom | A F G | A G | **A** F G |  |
| HLB | 0-10 cm mineral soil | 1.0 | Fly | **A** **F** G | A G | A F G |  |
| HLB | 0-10 cm mineral soil | 4.0 | Bottom | **A** F G | A G | A F G |  |
| HLB | 0-10 cm mineral soil | 4.0 | Fly | A F G | A **G** | **A** F **G** |  |
| ILK | 0-10 cm mineral soil | 1.4 | Bottom | A **F** G | A G | A F G | A F G |
| PLD | 0-10 cm mineral soil | 1.5 | Mixed | A F G | **A** G | A F G |  |
| SRD | 0-10 cm mineral soil | 1.0 | Fly | A F G | A G | A F **G** | A F G |
| HLB | FH-layer forest floor | 1.0 | Fly | **A** F G | A G | A F G | A F G |
| HLB | FH-layer forest floor | 4.0 | Bottom | **A** F G | A G | A F G |  |
| HLB | FH-layer forest floor | 4.0 | Fly | A F G | A G | **A** F G | A F G |
| HLB | FH-layer forest floor | 8.0 | Bottom | **A** F G | A G | A F G |  |
| HLB | FH-layer forest floor | 8.0 | Fly | A **F** **G** | A G | A F G | A F G |
| ILK | FH-layer forest floor | 0.7 | Bottom | A F **G** | A G | A F G | A F G |
| ILK | FH-layer forest floor | 2.8 | Bottom | A F **G** | A G | A F G | A F G |
| SRD | surface litter &/or moss with FH layer | 10.0 | Fly | **A** **F** G | A G | A F G | A F G |

# References

1.

Emilson, E. J. S. *et al.* Climate-driven shifts in sediment chemistry enhance methane production in northern lakes. *Nature Communications* **9**, 1801 (2018).

2.

Stoeck, T. *et al.* Multiple marker parallel tag environmental DNA sequencing reveals a highly complex eukaryotic community in marine anoxic water. *Mol Ecol* **19 Suppl 1**, 21–31 (2010).

3.

Folmer, O., Black, M., Hoeh, W., Lutz, R. & Vrijenhoek, R. DNA primers for amplification of mitochondrial cytochrome c oxidase subunit I from diverse metazoan invertebrates. *Mol Mar Biol Biotechnol* **3**, 294–299 (1994).

4.

Gibson, J. F. *et al.* Large-Scale Biomonitoring of Remote and Threatened Ecosystems via High-Throughput Sequencing. *PLOS ONE* **10**, e0138432 (2015).

5.

Menkis, A. *et al.* Occurrence and impact of the root-rot biocontrol agent Phlebiopsis gigantea on soil fungal communities in Picea abies forests of northern Europe. *FEMS Microbiology Ecology* **81**, 438–445 (2012).

6.

White, T. *et al.* Amplification and Direct Sequencing of Fungal Ribosomal RNA Genes for Phylogenetics. in *PCR Protocols: a Guide to Methods and Applications,* vol. 31 315–322 (1990).

7.

Oksanen, J. *et al.* *vegan: Community ecology package*. https://CRAN.R-project.org/package=vegan (2020).
